# Gene expression of single human mesenchymal stem cell in response to fluid shear

**DOI:** 10.1177/2041731412451988

**Published:** 2012-07-02

**Authors:** Hu Zhang, Alasdair Kay, Nicholas R Forsyth, Kuo-Kang Liu, Alicia J El Haj

**Affiliations:** 1 Institute of Science and Technology in Medicine, Keele University, Stoke-on-Trent, UK; 2 School of Chemical Engineering, The University of Adelaide, Adelaide, South Australia; 3 School of Engineering, The University of Warwick, Coventry, UK

**Keywords:** mesenchymal stem cell, fluid flow, shear stress, gene expression, osteogenic

## Abstract

Stem cell therapy may rely on delivery and homing through the vascular system to reach the target tissue. An optical tweezer model has been employed to exert different levels of shear stress on a single non-adherent human bone marrow–derived mesenchymal stem cell to simulate physiological flow conditions. A single-cell quantitative polymerase chain reaction analysis showed that collagen type 1, alpha 2 (*COL1A2*), heat shock 70-kDa protein 1A (*HSPA1A*) and osteopontin (*OPN*) are expressed to a detectable level in most of the cells. After exposure to varying levels of shear stress, there were significant variations in gene transcription levels across human mesenchymal stem cells derived from four individual donors. Significant trend towards upregulation of *COL1A2* and *OPN* gene expression following shear was observed in some donors with corresponding variations in *HSPA1A* gene expression. The results indicate that shear stress associated with vascular flow may have the potential to significantly direct non-adherent stem cell expression towards osteogenic phenotypic expression. However, our results demonstrate that these results are influenced by the selection process and donor variability.

## Introduction

Stem cell therapy is an emerging technique for the treatment and cure of many diseases for which adequate therapies do not exist, such as cardiac diseases^1^ and Parkinson’s disease.^2^ However, upon transplantation, stem cells and their derived lineages experience a multitude of biochemical and mechanical cues that influence cell behaviour.^3^ A fundamental understanding of the implications of the interplay of stem cell niche factors (growth factors, cell–cell contact and cell–matrix interaction) and external forces would enable the control of therapeutic cells and effective regeneration of functional tissues.^4^ An example of an evolving strategy for therapy would be the use of injectable solutions for stem cell treatments via intravenous injection into the body and delivery to target sites via the circulatory system. However, various safety concerns have been raised, which include the use of a heterogeneous population of cells, donor-to-donor variability, microbiological contamination, immunological response to alloantigens and tumorigenicity of the transplanted cells.^5^ It is therefore essential to develop standards and methodologies for characterizing products to address pre-clinical safety and efficacy.

One issue that arises when we discuss injectable approaches is the necessity to understand the effects of transport through the vascular system on phenotypic variation in autogenic and allogeneic sources of stem cells. In addition to this, the potential interactions between shear forces and cell behaviour and the ultimate differentiation capacity on delivery also need to be explored. Traditionally, stem cell population has been used for fluid-induced shear stress.^6–10^ However, adult stem cells are isolated with limited purity, even when the most advanced phenotypic marker combinations are utilized.^11,12^ Because of these variations, studies that are reliant on stem cell population are unable to accurately address many crucial biological and clinical questions.^13,14^ Analysing the cell population with single-cell precision may provide great insight into stem cell behaviours. In this study, a single cell from the human mesenchymal stem cell (hMSC) population will be used to examine the effect of shear stress on the stem cell.

Single-cell responses to mechanical stimulation can be characterized by a single-cell polymerase chain reaction (PCR). The major challenge for a single-cell PCR analysis is the small amounts of RNA obtained from an individual cell. Mammalian cells contain approximately 10–40 pg of RNA, of which about 0.1–1 pg is mRNA, corresponding to 10^5^–10^6^ messages.^15^ Recent studies have investigated the effects of compressive forces on a single cell and defined biomechanical profiles and molecular expression.^16^ There is, however, a lack of quantitative experimental models that enable effective study of the mechanical shear stresses on a single cell. Recently, optical tweezers have emerged as an essential tool for manipulating a single biological cell and performing sophisticated biophysical/biomechanical characterizations.^17^ In this study, we use optical tweezers to mimic the effects of blood circulation on non-adherent hMSCs during vascular delivery. Using this technique, we can exert varying levels of shear stress equivalent to physiological flow-induced stress on a single non-adherent stem cell. We have observed that the cells possessed a range of sizes from 20 to 40 μm. At higher velocity, some cells were visibly deformed under microscope analysis.^18,19^ The effects of shear stress on a profile of gene expressions were assessed using the single-cell quantitative PCR.

## Materials and methods

### Optical tweezers

Optical tweezers (Cell Robotics, Inc., Albuquerq, NM, USA), driven by a Windows XP–based Cell Robotic Workstation software, were used in the present experimental work. A Nd:YAG laser source was used at a wavelength of 1064 nm pumped by a 1.5-W diode. The laser was reflected through dichroic mirrors and focused through an inverted microscope (Nikon Optical Microsystem, Melville, NY, USA) before it reached the objects.

### Operational chambers

Two different chambers have been used for the experimental operation. A Lab-Tek chamber (Nunc Inc., Naperville, IL, USA) without coating was initially used for manipulation of hMSCs. The CoverWell perfusion chambers (Grace Bio-Labs, Inc., Bend, OR, USA) were coated with chemical reagents., To prevent adherence of stem cells on the surfaces of glass substrates, 40 mg poly(2-hydroxyethyl methacrylate) (Sigma, Dorset, UK) was dissolved in 2 mL of 95% ethanol. The thin cover glass was dipped into the solution and dried overnight at room temperature.

### Derivation of cells

hMSCs from four donors were obtained from Lonza (Walkersville, MD, USA) either as CD-selected population or as a bone marrow aspirate (BMA) (Table 1).

**Table 1.** Source and derivation of four populations of human bone marrow–derived MSCs for experimental analysis

	Age (years)	Sex
hBMA1	18	Male
hBMA2	21	Male
hBMA3	40	Male
CD_SEL	32	Male

MSCs: mesenchymal stem cells; hBMA: human bone marrow aspirate; CD_SEL: human Lonza CD selected.

#### CD-selected Lonza population

In the case of the CD-selected population, cells have been routinely characterized for positive expression of CD29, CD44, CD105 and CD106, and for negative expression of CD14, CD34 and CD45 by Lonza Biologics. For subsequent experimental procedure, CD-selected hMSCs were used at passage 5. Cells were resuspended in cell culture media (10% fetal calf serum (FCS), 1% antibiotics and 1% glutamine in Dulbecco’s modified Eagle’s medium (DMEM) solution (low glucose). The cells were placed in 24-well plates and maintained at 37°C in 5% CO_2_. The media were changed every 2 days, and the cells were monitored until cells became confluent. The cells were detached using trypsin–ethylenediaminetetraacetic acid (EDTA). After centrifugation, the cells were resuspended in serum-free media.

#### Isolation of hMSCs from BMA

hMSCs were isolated from commercially obtained BMA (Lonza) through an adhesion selection methodology.^20^ Mononuclear cells were plated at a density of 10^5^ cm^−2^ in 10 ng mL^−1^ fibronectin-coated (Sigma LS) in T75 flasks in high-glucose DMEM (Lonza) supplemented with 5% fetal bovine serum (FBS; Lonza), 1% non-essential amino acids (Invitrogen) and 1% l-glutamine (Invitrogen). After 7 days, half of the culture medium was removed and replaced with fresh one and after further 7 days, all media were removed. Isolated MSCs were subsequently cultured to confluency, passaged using standard procedures and multipotency confirmed through differentiation assays.^21^

### Cell manipulation and single-cell harvest

**Figure 1.**
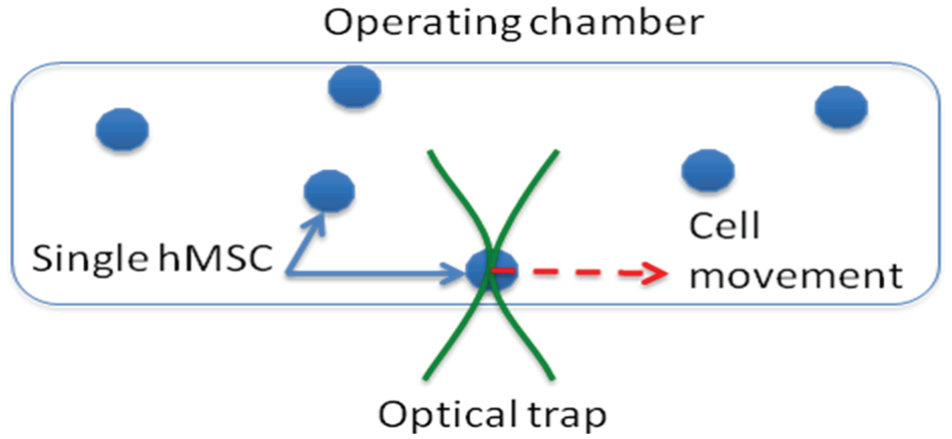
Schematic illustration of optical manipulation of a single hMSC in the operational chamber. Shear force is generated on the cell when the cell is forced to move in the liquid medium. hMSC: human mesenchymal stem cell.

On the day of experimentation, the cells were passaged using standard methodologies^21^ and diluted to around 1 × 10^3^ cells mL^−1^ in a serum-free medium. After gently shaking to keep cells uniformly distributed in the solution, 1 mL solution was pipetted into the surface-treated operational chamber. A 20× objective was used to screen an operational region where only one cell was present to avoid cell–cell contact during cell manipulation. After switching to the 100× objective, the laser was turned on to trap a single cell. The cell was moved in the operational chamber forward 0.5 mm and then backward 0.5 mm at different manipulation velocities as illustrated in Figure 1. After the manipulation, an aspirator tube controlled by a micromanipulator was carefully placed around the cell, and the pressure inside the tube was adjusted by a pneumatic actuator. The laser was turned off to release the cell. The cell was sucked into the aspirator tube by reducing the pressure in the tube. The volume of the solution containing the cell was kept less than 1 µL. The solution was transferred to a thin-walled RNase-free 0.5-mL PCR tube. This process was repeated for 9–12 cells in each sample grouping. To analyse the shear effect immediately after the manipulation, lysis buffer was added into the tube; and for the shear effect after 3–24 h, the solution containing the cell was placed into an incubator at 37°C with the lid closed.

### Molecular analysis

#### Cell lysis and reverse transcription

All reagents were supplied by Applied Biosystems/Ambion (Warrington, UK). Each cell was collected in 1 µL serum-free medium using a microaspirator and transferred to a thin-walled RNase-free 0.5-mL PCR tube containing 9 µL ice-cold cDNA II cell lysis buffer (Cells-to-cDNA™ II Kit; Ambion, CA, USA). Two negative controls were also processed: a medium blank control comprising 1 µL DMEM + 9 µL lysis buffer and a lysis blank control containing 10 µL lysis buffer. To rupture the cell, the mixture was incubated at 75°C for 10 min and then placed on ice. To degrade genomic DNA, the lysate was then treated with 0.2 µL RNase-free DNase I at 37°C for 15 min, followed by 75°C for 5 min. The RNA was reverse-transcribed in situ using a High Capacity cDNA RT kit. In brief, the following reagents were added to the entire volume of treated cell lysate (including the negative controls): 2 µL 10× reverse transcription (RT) buffer, 0.8 µL 25× deoxynucleotide triphosphates (dNTPs), 2 µL 10× random primers, 1 µL MultiScribe™ reverse transcriptase (50 U µL^−1^), 1 µL RNase inhibitor and 3.2 µL nuclease-free water. The mixture was incubated at 25°C for 10 min, followed by 37°C for 120 min. The reaction was terminated at 85°C for 5 s. A reverse transcriptase blank control was included, which contained kit reagents only.

#### cDNA preamplification and real-time quantitative PCR

All primer/probe mixtures were manufactured by Applied Biosystems. The specific TaqMan^®^ gene expression assay used was indicated for each gene studied. Each primer/probe mixture was supplied as a 20× concentrate. In total, nine genes of interest were studied (Table 2), including collagen type 1, alpha 2 (*COL1A2*, Hs00164099_m1), collagen type 2, alpha 1 (*COL2A1*, Hs00156568_m1), aggrecan (*ACAN*, Hs01048717_m1), osteopontin (*OPN*, Hs00167093_m1), alkaline phosphatase (*ALP*, Hs01029144_m1), heat shock 70-kDa protein 1A (*HSPA1A*, Hs00271229_s1), core-binding factor α-1 (*CBFA1* or *SOX9*, also known as runt-related transcription factors, *RUNX2*, Hs00231692_m1) and cyclooxygenase-2 (*COX2*, Hs00153133_m1). Gene expression was normalized to 18s rRNA. To amplify gene copy numbers to detectable levels, cDNA was first preamplified using TaqMan PreAmp Master Mix. In brief, all stock primer/probe mixes (except 18s rRNA) were prediluted together in Tris–EDTA buffer pH 8.0 to give 0.2× concentration. The preamplification reagent comprised 12.5 µL 2× TaqMan PreAmp Master Mix and 6.25 µL pooled 0.2× concentration. After adding 6.25 µL cDNA (or blank control), the tubes were placed in a PCR thermal cycler and incubated at 95°C for 10 min. Samples were preamplified for 14 cycles (denaturation at 95°C followed by annealing and extension at 60°C). The preamplified samples were diluted 1:20 with Tris–EDTA buffer. Gene expression was individually quantified for all genes of interest using the Applied Biosystems 7300 Real-Time PCR system. In brief, 12.5 µL TaqMan Universal PCR Master Mix, 1.25 µL 20× stock primer/probe mix and 18.75 µL nuclease-free H_2_O were added to each 6.25 µL preamplified, diluted cDNA sample or blank control.

**Table 2.** Levels of baseline gene expression in cultured hMSC (CD_SEL)

18s	*COL1A2*	*COL2A1*	*ALP*	*ACAN*	*OPN*	*HSPA*	*CBFA1*	*SOX9*	*COX2*
100%	92%	0%	4%	28%	46%	94%	19%	17%	25%

hMSC: human mesenchymal stem cell; CD_SEL: human Lonza CD selected.

### Relative abundance and statistical analysis

A relative abundance value of each gene of interest was calculated using a method adapted from Pfaffl.^22^ Briefly, the abundance, *A*, was calculated using the following equation

(1) $$A={\left(1+E\right)}^{-CT}$$

where *E* is the calculated amplification efficiency of the target gene and *CT* is the threshold cycle for that gene. PCR efficiency was calculated by running a standard curve for serially diluted cDNA prepared from hMSCs.

Means and standard deviation (SD) were calculated for all data sets. The statistical analysis was based on the GraphPad Prism 5.0. For the hMSCs isolated from BMA through the adhesion selection methodology, a regular two-way factorial analysis of variance (ANOVA) was preformed between group 1 of gene expression after 0 h and group 2 of gene expression after 18 h. A one-way ANOVA followed by Tukey’s test was performed for group 1 and group 2 individually. For CD-selected hMSCs, a one-way ANOVA followed by Tukey’s test was performed. Paired student’s *t*-test was used for gene expression after 0–24 h compared to the control group. The values of *p* lower than 0.05 were considered evidence for statistical significance.

### Theoretical analysis of shear stress on a single non-adherent cell

Individual cells were moved by the optical tweezers in the operational chamber to generate different velocities, resulting in the cells being subjected to different levels of shear stress. The force exerted on a single cell was calculated from the Stokes’ law, *F* = 6*πrηu*, where *r* is the cell radius, *η* is the liquid viscosity and *u* is the flow velocity. The shear stress was obtained by the force over the cross-sectional area of the single cell. The fluid velocity at 20, 40, 60 and 80 µm s^−1^ corresponds to a shear stress of 0.015, 0.030, 0.045 and 0.060 Pa, respectively.

## Results

### Gene expression of single hMSCs

To investigate the shear stress effect on hMSC gene expression, we examined nine genes: *COL1A2, COL2A1, ALP, ACAN, OPN, HSPA1A, CBFA1, SOX9* and *COX2* as well as the house-keeping gene, 18s rRNA, as indicated in Table 2. After 40 cycles of amplification, a threshold to eliminate background noise was applied to all samples on the same plate according to the user guide of Applied Biosystems (Warrington, UK). Of these, three genes, *COL1A2, OPN* and *HSPA1A*, achieved relative abundance levels of >40% and were selected for subsequent analysis. The current protocol of PCR analysis is still quite limited to the evaluation of very few, relatively highly expressed genes. Sensitive detection methods are being developed to improve the detectable levels of various genes.

### Responses of hMSCs (hBMA1–3) derived from different donors and CD-selected hMSCs

**Figure 2.**
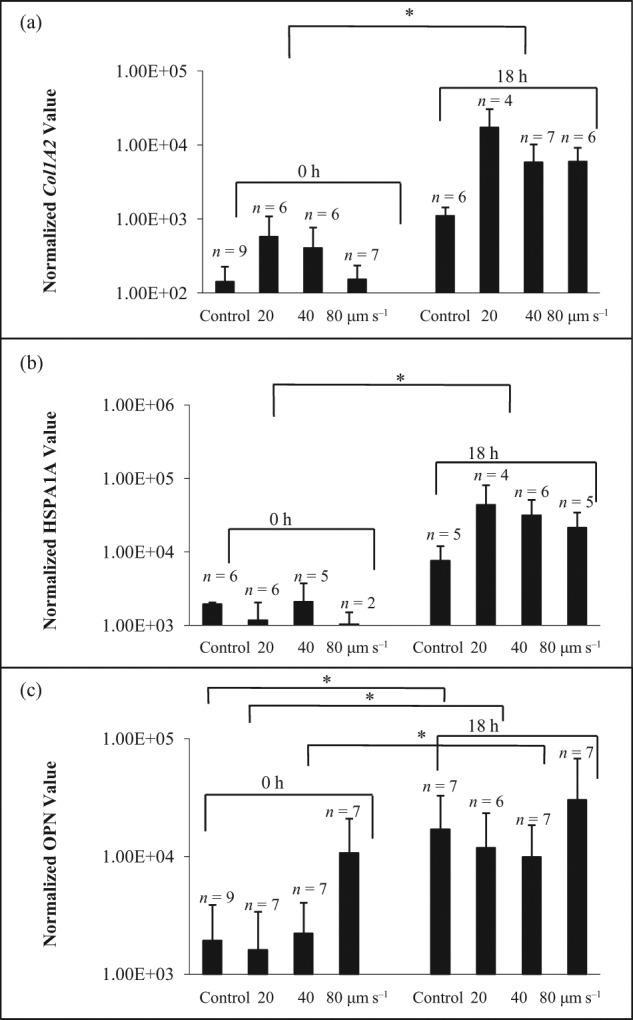
The effect of shear stress on gene expression of (a) *COL1A2*, (b) *HSPA1A* and (c) *OPN* for hMSC population hBMA1 (18-year-old male). The cells were lysed after collection at 0 and 18 h in the incubator. The data were normalized to 18s rRNA and presented as mean ± averaged absolute deviation. *n* means number of cells expressed out of 9–12 cells picked up after micromanipulation. The symbol ‘*’ indicates *p* < 0.05. hMSC: human mesenchymal stem cell.

**Figure 3.**
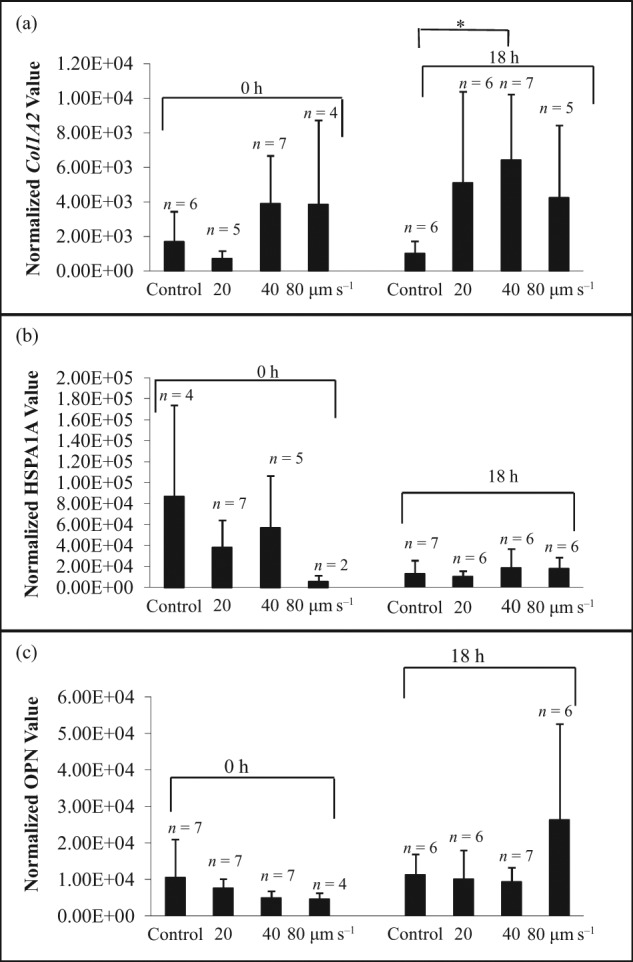
The effect of shear stress on gene expression of (a) *COL1A2*, (b) *HSPA1A* and (c) *OPN* for hMSC population hBMA2 (21-year-old male). The cells were lysed after collection at 0 and 18 h in the incubator. The data were normalized to 18s rRNA and presented as mean ± averaged absolute deviation. *n* means number of cells expressed out of 9–12 cells picked up after micromanipulation. The symbol ‘*’ indicates *p* < 0.05 hMSC: human mesenchymal stem cell.

**Figure 4.**
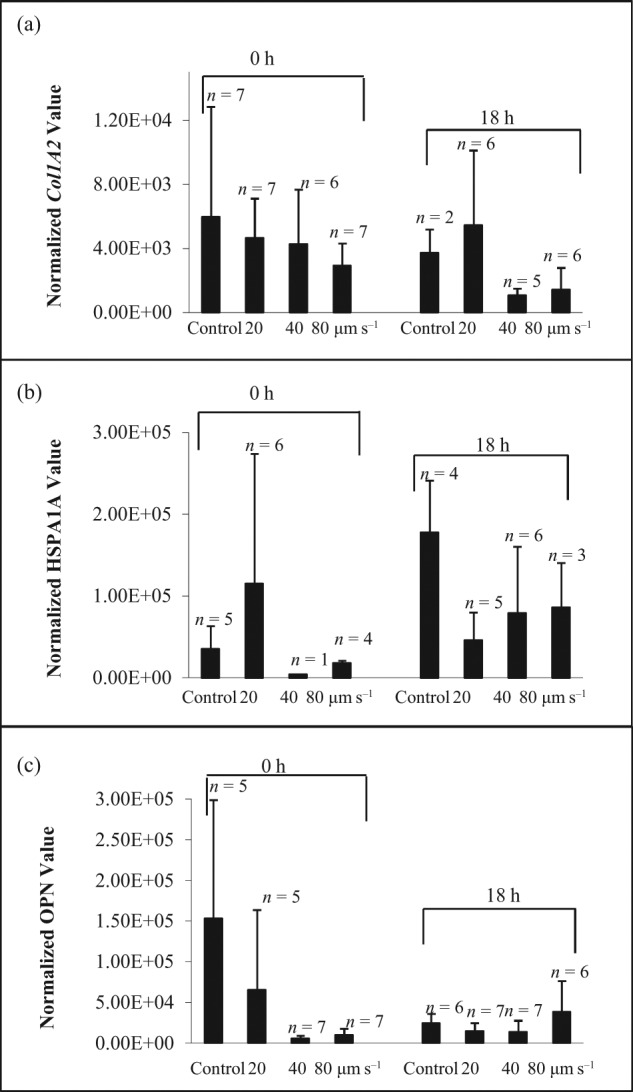
The effect of shear stress on gene expression of (a) *COL1A2*, (b) *HSPA1A* and (c) *OPN* for hBMSC population hBMA3 (40-year-old male). The cells were lysed after collection at 0 and 18 h in the incubator. The data were normalized to 18s rRNA and presented as mean ± averaged absolute deviation. *n* means number of cells expressed out of 9–12 cells picked up after micromanipulation. hBMSC: human bone marrow–derived mesenchymal stem cell.

The effects of shear stress on hMSC expression of the genes, *COL1A2, HSPA1A* and *OPN*, from three individual donors are shown in Figures 2 to 4. All donor-derived hMSCs demonstrated individual-specific sensitivity to the manipulating velocities. For hBMA1, *COL1A2* expression (Figure 2(a)) was significantly increased (*p* < 0.05) after manipulating at 20, 40 and 80 µm s^−1^. *HSPA1A* expression (Figure 2(b)) after 18 h was also markedly increased (*p* < 0.05) after 20, 40 and 80 µm s^−1^ manipulation. When lysing cells immediately after collection, *OPN* expression (Figure 2(c)) was significantly increased (*p* < 0.05) after manipulating at 20 and 40 µm s^−1^. In contrast, there was no significant difference in *HSPA1A* (Figure 3(b)) and *OPN* gene expression (Figure 3(c)) at 0 and 18 h through all manipulating velocities in patient hBMA2. Although *COL1A2* expression (Figure 3(a)) showed an increase in response to shear at 18 h compared to control at 18 h, the difference was only significant between control and 40 µm s^−1^. In contrast with hBMA3, the effect of shear stress on gene expression for *COL1A2* (Figure 4(a)), *HSPA1A* (Figure 4(b)) and *OPN* (Figure 4(c)) was not significant after 0 and 18 h.

**Figure 5.**
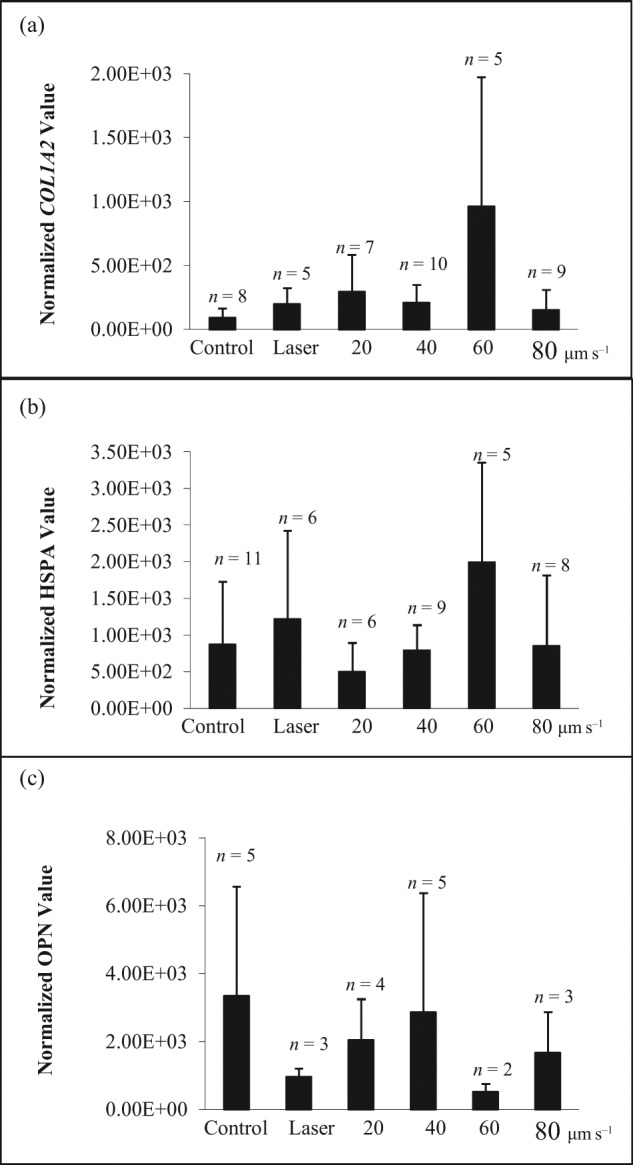
The expression of (a) *COL1A2*, (b) *HSPA1A* and (c) *OPN* of CD-selected population (passage 5) after exposing to different levels of shear stress. The second bar represents the cells exposed to laser heating only. The single cell was lysed after collection. The data were normalized to 18s rRNA and presented as mean ± averaged absolute deviation. *n* means number of cells expressed out of 9–12 cells picked up after micromanipulation.

In the population of CD-selected cells from Lonza grown in culture to passage 5, *COL1A2* expression (Figure 5(a)) was elevated in response to shear stress of 60 µm s^−1^ when compared to control samples. However, the increase at shear stress levels at a manipulation velocity ranging from 20 to 80 µm s^−1^ was not significant when compared to control samples. Expression of *HSPA1A* (Figure 5(b)) and *OPN* (Figure 5(c)) was not elevated in response to increasing levels of shear in CD-selected hMSCs.

**Figure 6.**
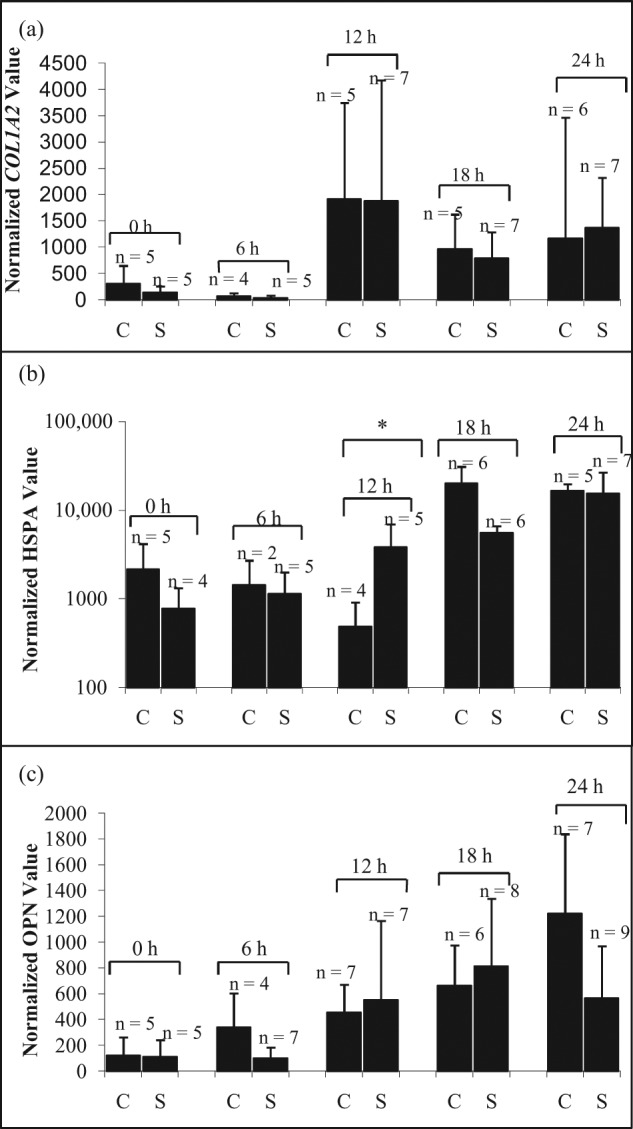
The effect of incubation time before cell lysis on gene expression of (a) *COL1A2*, (b) *HSPA* and (c) *OPN* after CD-selected cells is manipulated at 40 µm s^−1^. The cells were lysed after collection at 0, 6, 12, 18 and 24 h in the incubator. The data were normalized to 18s rRNA and presented as mean ± averaged absolute deviation. *n* means number of cells expressed. The symbol ‘*’ indicates *p* < 0.05. C: control; S: shear.

The gene expression profiles for different durations following application of shear to CD-selected hMSCs were then examined to determine if fluctuations were occurring over a wider time range. Following manipulation at 40 µm s^−1^, each single cell was suspended at 37°C in serum-free media for 0, 6, 12, 18 and 24 h. No significant effect of shear stress on gene expression of *COL1A2* (Figure 6(a)), *OPN* (Figure 6(c)) and *HSPA1A* (Figure 6(b)) was observed, except following 12-h incubation where *HSPA1A* expression (Figure 6(b)) was significantly increased (*p* < 0.05).

## Discussion

The average levels of venous and arterial wall shear stresses range between 0.1–0.5 Pa and 0.6–4.0 Pa, respectively.^23^ However, the shear stress acting on a single suspension hMSC is below the physiological wall shear stress. The calculated shear stress in venous flow is around 0.004–0.08 Pa. The shear stress exerting on a single hMSC by optical tweezers at a flow velocity from 20 to 80 µm s^−1^ was calculated to be 0.015–0.06 Pa, which falls within the physiological flow range.

In our study, mechano-inductive gene expression was upregulated in response to varying levels of stress. These responses were not consistent across all four donors. A reduction in response was also noted with an ageing source of patient material although there may be other factors which explain these responses. In addition, CD-selected hMSC after extended growth in culture showed a decline in shear response as has been observed for adherent selected cells at an earlier stage of culture/passage.^24^ These variations demonstrate the inherent variability in using mixed populations of bone marrow–derived cells with quite marked cell responses to environmental cues; a feature which must be considered when designing cell therapies and sources of cells for use in therapeutic treatments.

### Analysis of HSPA1A expression

*HSPA1A*, heat shock 70-kDa protein 1A, is involved in cell protection from stress and apoptosis. It has been reported that shear stress could result in the induction of genes involved in mediating the cellular response to stress including *HSPA1A*.^25^ However, this gene may also be induced by the stress from the heating and photodamage to stem cells due to laser absorption. Optical tweezers generate a highly focused spot with power intensities of megawatts per square centimetre,^26^ which can lead to damage or ‘opticution’ due to laser adsorption. Laser-induced effects on cell viability, growth and division have been found to be significant in *Escherichia coli*^27^ and Chinese hamster ovary (CHO) cells.^28^ *HSPA1A* could be induced by either shear stress effect or photodamaging effect. To differentiate between these two effects, the hMSCs were exposed to the laser source only without any movement for 5–10 min. There was no significant increase in response in all cell types in response to the laser alone as shown in Figure 5. A possible explanation is that near-infrared laser Nd:YAG (1064 nm in wavelength) was chosen as a lower energy source to mitigate the photodamaging effects of optical tweezers.

Our observations illustrate that hMSC responses to shears stress were largely donor specific. *HSPA1A* expression was significantly increased in hBMA1 after shear stress, respectively. However, upregulation of *HSPA1A* was not consistently observed. Expression levels of *HSPA1A* were either largely unchanged (hBMA2) or reduced in response to low levels of shear stress (hBMA3). The mechanisms remain to be clarified through further studies.

### COL1A2 and OPN expression

hMSCs derived from adherent selection demonstrated a tendency towards osteogenic differentiation after fluid shear stress exposure. Both collagen I and osteopontin are key markers of osteogenic lineage differentiation. In addition, osteopontin has been shown to be an early marker of mechanical induction.^29^ Osteogenic induction as a shear stress response agrees with previous data on adherent monolayer and three-dimensional (3D) cultures of MSC exposed to varying levels of shear.^6–10^

Similar to *HSPA1A*, we have noted donor-specific responses in expression levels of *COL1A2* and *OPN* after shear stress manipulation. *COL1A2* expression was increased after 0.030 Pa shear stress exposure for both hBMA1 and hBMA2 but not hBMA3. Upregulation of *COL1A2* was also noted for CD_SEL. Common upregulation of *OPN* was also noted for both hBMA1 and hBMA2 at 80 µm s^−1^, while reduced or control level expression of *OPN* was noted for both hBMA3 and CD_SEL.

Although we have found that some statistically significant changes in gene expression are donor specific after exposing stem cells to shear stress, it is important to note that a significant number of hMSCs remain untouched when circulating inside the human body in the physiological conditions. However, in this condition, cells are smoothly flown in the blood vessel without considering the effect of cell rotation and adhesion. It has been demonstrated that characterization of the population at the single-cell level shows a very variable population. More work needs to be carried out to understand the characteristics of stem cells and the potential impact of delivery to the site of repair.

## Conclusions

A single-cell approach for studying fluid mechanical influence on gene expression of hMSCs has been developed based on a unique combination of optical tweezers and single-cell PCR. Optical tweezers have been used to apply different levels of shear stress to hMSCs which correspond to physiological stress levels observed during vascular flow.

The effect of shear stress has been examined by single-cell PCR analysis of nine genes (Table 2). Among these, only *COL1A2, HSPA1A* and *OPN* were expressed to robustly determinable levels. Transcriptional responses to shear stress applications were noted which encompassed both upregulation and downregulation of the same gene in an individual-specific manner. This single-cell PCR analysis combined with optical tweezers could be used to understand the fundamental stem cell biology and formulate the strategies for stem cell delivery.
